# Supplementary material for: Albuminuria Changes as a surrogate endpoint in Apolipoprotein L1 Mediated Kidney Disease in Vanderbilt BioVU and the Million Veteran Program
Source: medRxiv. 2026 Jun 8:2026.06.04.26354945. Preprint. [Version 1] doi: 10.64898/2026.06.04.26354945 (PMC13278290; doi:10.64898/2026.06.04.26354945)
Supplement: Supplement 1 [file NIHPP2026.06.04.26354945v1-supplement-1.pdf]

medRxiv preprint doi: https://doi.org/10.64898/2026.06.04.26354945; this version posted June 8, 2026. The copyright holder for this preprint (which was not certified by peer review) is the author/funder, who has granted medRxiv a license to display the preprint in perpetuity.
This article is a US Government work. It is not subject to copyright under 17 USC 105 and is also made available for use under a CC0 license.

Supplemental material

Supplemental Figure 1. Study design

Supplemental Figure 2. Flow chart for sensitivity analysis for patients with baseline UACR ≥ 321 mg/g

Supplementary Table 1. Clinical characteristics for sensitivity analysis baseline UACR ≥ 321 mg/g

Supplementary Table 2. Association between log-UACR changes from baseline to 12 months and eGFR slope over 24 months among non-diabetic patients with 2 APOL1 high risk variants with baseline UACR ≥ 321 mg/g

Supplementary Table 3. The effect of UACR change at 12 months on the risk of the clinical meaningful endpoint among non-diabetic proteinuric patients with 2 APOL1 high risk variants with baseline UACR ≥ 321 mg/g

Supplementary Table 4. MVP Core acknowledgement

medRxiv preprint doi: https://doi.org/10.64898/2026.06.04.26354945; this version posted June 8, 2026. The copyright holder for this preprint (which was not certified by peer review) is the author/funder, who has granted medRxiv a license to display the preprint in perpetuity.
This article is a US Government work. It is not subject to copyright under 17 USC 105 and is also made available for use under a CC0 license.

Supplemental Figure 1. Study design

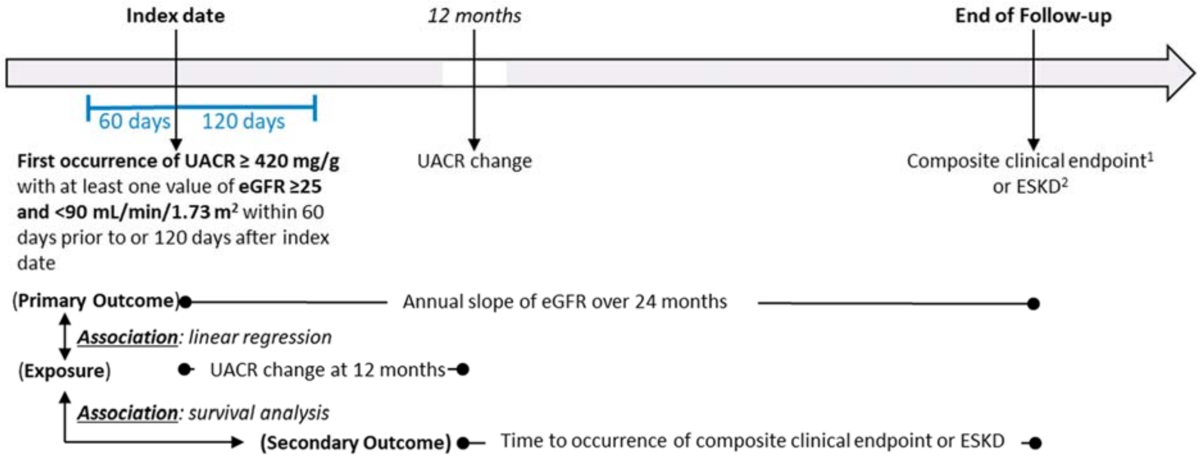

[1] *Composite clinical endpoint*: earliest occurrence of ESKD, sustained ≥30% eGFR decline (at least two ≥30% declines occurred at least 28 days apart), or death
[2] *ESKD*: earliest occurrence of sustained eGFR < 15 mL/min/1.73m² (at least 2 measurements taken at least 28 days apart), dialysis, or transplantation

medRxiv preprint doi: https://doi.org/10.64898/2026.06.04.26354945; this version posted June 8, 2026. The copyright holder for this preprint (which was not certified by peer review) is the author/funder, who has granted medRxiv a license to display the preprint in perpetuity. This article is a US Government work. It is not subject to copyright under 17 USC 105 and is also made available for use under a CC0 license.

Supplemental Figure 2. Sensitivity analysis:

Flow chart for patients with baseline UACR ≥ 321 mg/g

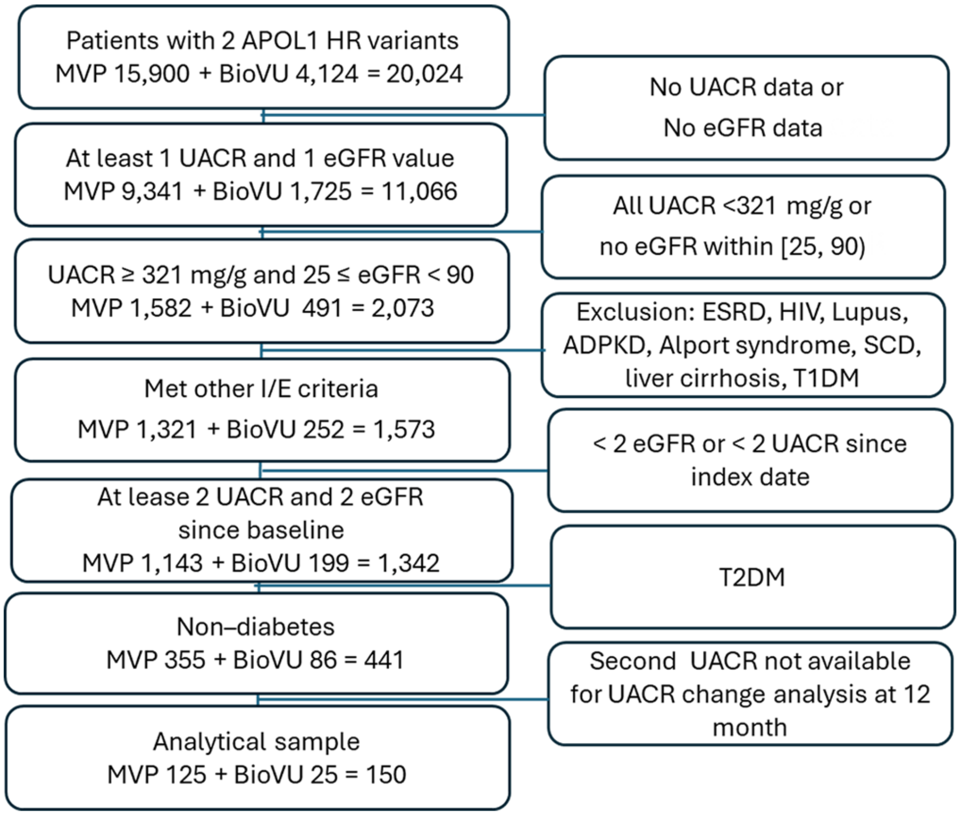

medRxiv preprint doi: https://doi.org/10.64898/2026.06.04.26354945; this version posted June 8, 2026. The copyright holder for this preprint (which was not certified by peer review) is the author/funder, who has granted medRxiv a license to display the preprint in perpetuity.
This article is a US Government work. It is not subject to copyright under 17 USC 105 and is also made available for use under a CC0 license.

Supplemental Table 1. Clinical characteristics for cohort in sensitivity analysis 2 (i.e., baseline UACR ≥ 321 mg/g, 25 ≤ baseline eGFR0 < 90)

	BioVU	MVP	Pooled
No. patients	25	125	150
Age, years (mean, SD)	47.9 (17.9)	58.6 (14.8)	56.8 (15.8)
Males (n, %)	15 (60.0%)	118 (94.4%)	133 (88.7%)
eGFR, mL/min/1.73m^2 (mean, SD)	48.1 (19.0)	46.7 (16.6)	46.9 (17.0)
UACR, mg/g (mean, SD)	1275.9 (1388.2)	997.8 (1014.7)	1044.2 (1085.4)
ACEi or ARBs (n, %)	10 (40.0%)	94 (75.2%)	104 (69.3%)
SGLT2 (n, %)	0 (0.0%)	1 (0.8%)	1 (0.7%)
FSGS, baseline (n, %)	3 (12.0%)	0 (0.0%)	3 (2.0%)
FSGS, within follow up (n, %)	3 (12.0%)	7 (5.6%)	10 (6.7%)
Follow-up, yrs (mean, SD)	3.7 (4.4)	3.5 (3.6)	3.5 (3.7)
Follow-up, yrs (median, IQR)	1.8 [0.8;5.5]	2.6 [1.1;4.3]	N/A*
Annual eGFR slope over 24 months (mL/min/1.73m2/year), 95% CI	-6.56 [-13.18, 0.06]	-3.60 [-5.03, -2.17]	-3.73 [-5.13, -2.33]
Geometric mean percentage change in proteinuria at 12 months (%, 95% CI)	-51.1% [-70.6%, -18.7%]	-52.7% [-61.5%, -41.9%]	-52.5% [-60.6%, -42.6%]
Meaningful clinical endpoints			
30% decline in eGFR	14 (56.0%)	78 (62.4%)	92 (61.3%)
End stage kidney disease	12 (48.0%)	52 (41.6%)	64 (42.7%)
death	3 (12.0%)	39 (31.2%)	42 (28.0%)
Composite	15 (60.0%)	88 (70.4%)	103 (68.7%)

N/A*: Not Available, since there is no feasible approach to get pooled median IQR without combining individual data.

medRxiv preprint doi: https://doi.org/10.64898/2026.06.04.26354945; this version posted June 8, 2026. The copyright holder for this preprint (which was not certified by peer review) is the author/funder, who has granted medRxiv a license to display the preprint in perpetuity.
This article is a US Government work. It is not subject to copyright under 17 USC 105 and is also made available for use under a CC0 license.

Supplemental Table 2. Association between log-UACR changes from baseline to 12 months and eGFR slope over 24 months among non-diabetic patients with 2 APOL1 high risk variants for patients with baseline UACR ≥ 321 mg/g

	BioVU		MVP		Pooled	
	b [95% CI]	*p*	*b [95% CI]*	*p*	*b [95% CI]*	*p*
Sensitivity analysis (baseline UACR ≥ 321 mg/g; 25 ≤ baseline eGFR < 90)						
Model 1	-1.38 [-5.96, 3.20]	0.54	-1.23 [-2.10, -0.37]	0.005	-1.24 [-2.08, -0.40]	0.004
Model 2	-1.75 [-6.62, 3.12]	0.46	-1.31 [-2.18, -0.44]	0.003	-1.32 [-2.17, -0.48]	0.002
Model 3	-2.88 [-8.15, 2.39]	0.27	-1.31 [-2.19, -0.43]	0.004	-1.36 [-2.22, -0.50]	0.002
Sensitivity analysis (baseline UACR ≥ 321 mg/g; 25 ≤ baseline eGFR < 75)						
Model 1	-1.44 [-6.12, 3.24]	0.53	-1.56 [-2.39, -0.73]	<0.001	-1.56 [-2.36, -0.75]	<0.001
Model 2	-1.86 [-6.86, 3.13]	0.44	-1.60 [-2.43, -0.78]	<0.001	-1.61 [-2.42, -0.81]	<0.001
Model 3	-2.04 [-7.07, 2.98]	0.40	-1.63 [-2.46, -0.79]	<0.001	-1.64 [-2.45, -0.83]	<0.001

medRxiv preprint doi: https://doi.org/10.64898/2026.06.04.26354945; this version posted June 8, 2026. The copyright holder for this preprint (which was not certified by peer review) is the author/funder, who has granted medRxiv a license to display the preprint in perpetuity.
This article is a US Government work. It is not subject to copyright under 17 USC 105 and is also made available for use under a CC0 license.

Supplemental Table 3. The effect of UACR change at 12 months on the risk of the clinical meaningful endpoint among non-diabetic proteinuric patients with 2 APOL1 high risk variants

	BioVU		MVP		Pooled	
	HR [95% CI]	*p*	*HR [95% CI]*	*p*	*HR [95% CI]*	*p*
Clinical Composite Outcome						
Sensitivity analysis (baseline UACR ≥ 321 mg/g; 25 ≤ baseline eGFR < 90)						
Model 1	1.94 [0.91, 4.16]	0.09	1.39 [1.08, 1.78]	0.01	1.43 [1.13, 1.81]	0.003
Model 2	1.91 [0.86, 4.20]	0.11	1.41 [1.09, 1.82]	0.008	1.45 [1.14, 1.85]	0.003
Model 3	1.63 [0.72, 3.68]	0.24	1.41 [1.09, 1.83]	0.009	1.43 [1.12, 1.83]	0.005
Sensitivity analysis (baseline UACR ≥ 321 mg/g; 25 ≤ baseline eGFR < 75)						
Model 1	1.86 [0.85, 4.06]	0.12	1.40 [1.08, 1.81]	0.01	1.44 [1.12, 1.83]	0.004
Model 2	1.71 [0.77, 3.79]	0.19	1.43 [1.09, 1.86]	0.009	1.45 [1.13, 1.87]	0.004
Model 3	1.89 [0.82, 4.36]	0.14	1.44 [1.10, 1.88]	0.009	1.47 [1.14, 1.91]	0.003
End Stage Kidney Disease						
Sensitivity analysis (baseline UACR ≥ 321 mg/g; 25 ≤ baseline eGFR < 90)						
Model 1	3.23 [1.30, 8.02]	0.01	1.68 [1.21, 2.32]	0.002	1.81 [1.33, 2.45]	<0.001
Model 2	4.23 [1.16, 15.43]	0.03	1.71 [1.22, 2.38]	0.002	1.81 [1.31, 2.49]	<0.001
Model 3	4.88 [1.10, 21.63]	0.04	1.71 [1.22, 2.40]	0.002	1.80 [1.29, 2.51]	<0.001
Sensitivity analysis (baseline UACR ≥ 321 mg/g; 25 ≤ baseline eGFR < 75)						
Model 1	3.15 [1.29, 7.69]	0.01	1.73 [1.24, 2.40]	0.001	1.85 [1.36, 2.52]	<0.001
Model 2	4.91 [1.19, 20.20]	0.03	1.78 [1.27, 2.51]	0.001	1.89 [1.35, 2.63]	<0.001
Model 3	5.21 [1.22, 22.26]	0.03	1.80 [1.27, 2.54]	0.001	1.90 [1.36, 2.66]	<0.001

N/A*: Not Available, since the model fitting cannot converge to estimate the HR and 95% CI.

medRxiv preprint doi: https://doi.org/10.64898/2026.06.04.26354945; this version posted June 8, 2026. The copyright holder for this preprint (which was not certified by peer review) is the author/funder, who has granted medRxiv a license to display the preprint in perpetuity. This article is a US Government work. It is not subject to copyright under 17 USC 105 and is also made available for use under a CC0 license.

Supplemental Table 4. MVP Core Acknowledgements for Publications_October 2025

VA Million Veteran Program
Core Acknowledgements for Publications
October 2025

MVP Program Office
- Sumitra Muralidhar, Ph.D., Program Director
US Department of Veterans Affairs, 810 Vermont Avenue NW, Washington, DC 20420
- Jennifer Moser, Ph.D., Associate Director, Scientific Programs
US Department of Veterans Affairs, 810 Vermont Avenue NW, Washington, DC 20420
- Jennifer E. Deen, B.S., Associate Director, Cohort & Public Relations
US Department of Veterans Affairs, 810 Vermont Avenue NW, Washington, DC 20420

MVP Steering Committee
- Co-Chair: Philip S. Tsao, Ph.D.
VA Palo Alto Health Care System, 3801 Miranda Avenue, Palo Alto, CA 94304
- Co-Chair: Sumitra Muralidhar, Ph.D.
US Department of Veterans Affairs, 810 Vermont Avenue NW, Washington, DC 20420
- J. Michael Gaziano, M.D., M.P.H.
VA Boston Healthcare System, 150 S. Huntington Avenue, Boston, MA 02130
- Adriana Hung, M.D., M.P.H.,
VA Tennessee Valley Healthcare System, 1310 24th Avenue, South Nashville, TN 37212
- Dave Oslin, M.D.
Philadelphia VA Medical Center, 3900 Woodland Avenue, Philadelphia, PA 19104
- Deepak Voora, M.D.
Durham VA Medical Center, 508 Fulton Street, Durham, NC 27705

MVP Co-Principal Investigators
- J. Michael Gaziano, M.D., M.P.H.
VA Boston Healthcare System, 150 S. Huntington Avenue, Boston, MA 02130
- Philip S. Tsao, Ph.D.
VA Palo Alto Health Care System, 3801 Miranda Avenue, Palo Alto, CA 94304

MVP Core Operations
- Jessica V. Brewer, M.P.H., Director, MVP Cohort Operations
VA Boston Healthcare System, 150 S. Huntington Avenue, Boston, MA 02130
- Mary T. Brophy M.D., M.P.H., Director, VA Central Biorepository
VA Boston Healthcare System, 150 S. Huntington Avenue, Boston, MA 02130
- Kelly Cho, M.P.H, Ph.D., Director, MVP Phenomics
VA Boston Healthcare System, 150 S. Huntington Avenue, Boston, MA 02130
- Lori Churby, B.S., Director, MVP Regulatory Affairs
VA Palo Alto Health Care System, 3801 Miranda Avenue, Palo Alto, CA 94304
- Jacob T. Kean, Ph.D., Acting Director, VA Informatics and Computing Infrastructure (VINCI)
VA Salt Lake City Health Care System, 500 Foothill Drive, Salt Lake City, UT 84148
- Saiju Pyarajan Ph.D., Director, Data and Computational Sciences

medRxiv preprint doi: https://doi.org/10.64898/2026.06.04.26354945; this version posted June 8, 2026. The copyright holder for this preprint (which was not certified by peer review) is the author/funder, who has granted medRxiv a license to display the preprint in perpetuity. This article is a US Government work. It is not subject to copyright under 17 USC 105 and is also made available for use under a CC0 license.

VA Boston Healthcare System, 150 S. Huntington Avenue, Boston, MA 02130
- Robert Ringer, Pharm.D., Director, VA Albuquerque Central Biorepository
New Mexico VA Health Care System, 1501 San Pedro Drive SE, Albuquerque, NM 87108
- Luis E. Selva, Ph.D., Director, MVP Biorepository Coordination
VA Boston Healthcare System, 150 S. Huntington Avenue, Boston, MA 02130
- Shahpoor (Alex) Shayan, M.S., Director, MVP PRE Informatics
VA Boston Healthcare System, 150 S. Huntington Avenue, Boston, MA 02130
- Brady Stephens, M.S., Principal Investigator, MVP Information Center
Canandaigua VA Medical Center, 400 Fort Hill Avenue, Canandaigua, NY 14424
- Stacey B. Whitbourne, Ph.D., Director, MVP Cohort Development and Management
VA Boston Healthcare System, 150 S. Huntington Avenue, Boston, MA 02130 2.
